# Middle-aged women with hematodiaphyseal dysplasia: Ghosal syndrome: Case report

**DOI:** 10.1016/j.radcr.2024.07.028

**Published:** 2024-08-06

**Authors:** Diviya Bharathi Ravikumar, Barath Prashanth Sivasubramanian, Shreya Thungala, Gopinath Srinivasan, Abul Hasan Shadali Abdul Khader, Husna Qadeer, Viraj Panchal, Vikram Samala Venkata

**Affiliations:** a Department of Internal Medicine, ESIC MC and PGIMSR, Chennai, Tamil Nadu, India; b Department of Infectious Diseases, University of Texas Health, San Antonio, TX, USA; c Department of Radiology, ESIC MC and PGIMSR, Chennai, Tamil Nadu, India; d Department of Internal Medicine, Government Kilpauk Medical College, Chennai, Tamil Nadu, India; e Department of Internal Medicine, Smt. N.H.L. Municipal Medical College and SVPISMR, Ahmedabad, Gujarat, India; f Hospital Medicine Division, Cheshire Medical Center/Dartmouth Hitchcock Keene, NH, USA

**Keywords:** Ghosal syndrome, Ghosal hematodiaphyseal dysplasia, Anemia, Bone pain, Metadiaphyseal dysplasia, Bone marrow hypoplasia

## Abstract

Ghosal hematodiaphyseal dysplasia (GHDD) is a rare autosomal recessive disorder characterized by increased bone density involving diaphyses of long bones and defective hematopoiesis. It is due to biallelic variants in the TBXAS1 (OMIM*274180) gene, which encodes for thromboxane synthase. We present a rare case of a middle-aged woman who presented with chronic anemia and bone pain. About 31-year-old Southeast Asian female with a history of persistent iron deficiency anemia (6.1 gm/dL) presents with bilateral knee pain for 4 years. Autoimmune panel turned out to be negative. CT scan of the lower limbs showed multilamellated endosteal thickening specifically involving diaphyses with severe narrowing of medullary canal. PET CT scan revealed tubular remodeling, intramedullary ground glass matrix, and mild cortical thickening with increased FDG uptake in diaphyseal regions of femur and tibia. Bone marrow biopsy of left tibia revealed fibrocellular marrow with dyserythropoiesis. Considering the slow progression of illness over 4 years and radiological evidence suggestive of bone remodeling with severe narrowing of medullary canal as the cause of anemia, the patient underwent molecular analysis for GHDD. Results revealed homozygous p.Arg412Gln (exon 11) in TBXAS1 gene. Considering the effect of NSAIDs on cyclooxygenase and its downstream metabolites, oral Aspirin 150 mg/day was initiated. Hemoglobin improved to 11 gm/dL at 3-month follow-up visit. The complexity of reaching a diagnosis of GHDD underscores the importance of maintaining a high clinical suspicion and thorough analysis of radiological evidence. The treatment for GHDD involves aspirin, a readily available drug.

## Introduction

Ghosal hemato-diaphyseal dysplasia (GHDD; OMIM #231095), also known as Ghosal syndrome, was first described in 1988 [1]. It is a rare autosomal recessive disorder, characterized by myelophthisic anemia and distinctive bony changes, including metadiaphyseal cortical thickening on imaging [2]. Less than 40 cases have been reported worldwide, of which 17 cases are from the Middle East and Southeast Asia, suggesting a common racial background and probably a genetic pool [3,4]. Most GHDD cases observed in pediatric population; only 2 adult cases have been reported. Our case is the third globally documented occurrence in adults, the second in Southeast Asia [5,6].

The underlying mechanisms of GHDD remain unclear [4]. GHDD is attributed to biallelic pathogenic variants or missense mutations in TBXAS1 gene (chromosome 7q33-34) [6,7]. This gene encodes for thromboxane synthase, an enzyme participating in the arachidonic acid cascade [7]. Thromboxane A2 (TXA2) modulates the expression of TNFSF11 and TNFRSF11B, which encode RANKL and osteoprotegerin (OPG) in osteoblasts and it also induces osteoclast formation. TXA2 thereby regulates bone mineral density and dysregulation of TXA2 promotes sclerosis [8]. Secondly, dysregulation of TXA2 leads to an increase in prostaglandin H2 production. This, in turn, results in the accumulation of downstream proinflammatory prostaglandins and leukotriene metabolites. These metabolites may cause direct injury to the bone marrow, leading to cortical remodeling [4]. Abnormal sclerosis causes marrow hypocellularity eventually [6]. GHDD presents with moderate to severe anemia, severe pancytopenia, with or without splenomegaly, myelofibrosis, and diaphyseal dysplasia mostly presented with endosteal thickening of long bones and widening of metadiaphysis [9,10]. Here, we present a case of adult-onset GHDD.

## Case presentation

**Figure 1:**
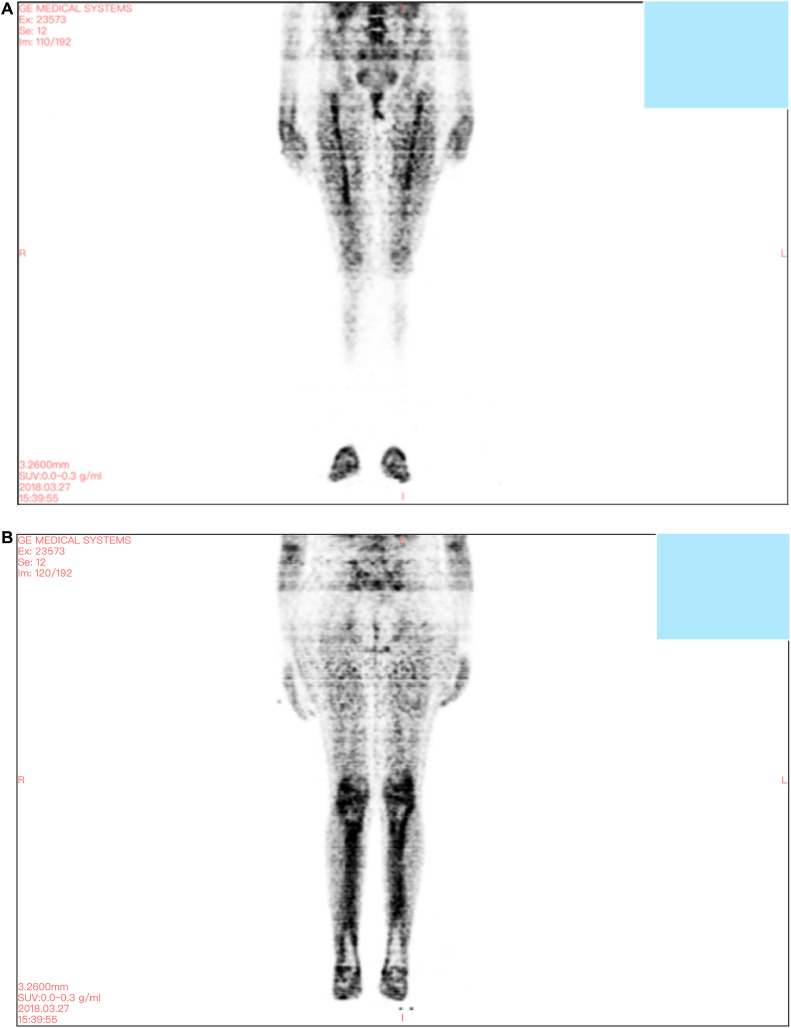
A,B. PET CT scan demonstrating increased FDG uptake in bilateral distal femoral and proximal tibial epiphyseal region and diaphyseal region.

A 31-year-old female of Southeast Asian descent, born to consanguineous parents, presented to the clinic with complaints of bilateral knee pain, swelling, mild leg bowing, and a 4-year history of chronic anemia with hemoglobin of 6.1 g/dL. Patient was earlier diagnosed with iron deficiency anemia and treated with multiple iron ± blood transfusions. No comprehensive workup has been done to date. The persistence of her symptoms over a 4-year period prompted further evaluation. Hemoglobin electrophoresis and hemolytic panel did not yield any significant results. Autoimmune panel for rheumatoid arthritis was negative. MRI of both the knees showed periarticular soft tissue edema with tubular appearance of the distal femur and proximal tibia with irregular large areas of hypointensities along with right tibial upper metadiaphyseal small sclerotic foci. Due to the identification of sclerotic foci, the radiologist suggested a PET CT scan and a correlation of the results with a bone biopsy. PET CT scan showed tubular remodeling, intramedullary ground glass matrix, and mild cortical thickening with increased FDG uptake in bilateral distal femoral and proximal tibial epiphyseal region, and diaphyseal region (Fig. 1).

Following this, bone marrow aspiration cytology from left anterior tibia revealed bone marrow diluted with peripheral blood, with a reduction in all 3 cell progenitors. Additionally, bone marrow biopsy of the same showed osteosclerosis with fibrocellular marrow. Hematopoietic elements showed dyserythropoiesis, dysmegakaryopoiesis, and variable lymphohistiocytic infiltrate.

The results pointed toward disorders causing cortical thickening and bone marrow sclerosis. Patient was tested for myelofibrosis but was found to be triple negative for the disease (JAK2 V617F (exon12) and JAK2 exon 14 mutations, exon 9 of CALR, and exon 10 of MPL). A provisional diagnosis of Erdheim Chester Disease (ECD) was made in view of histological and radiological findings. Immunohistochemistry for CD 68 showed few scattered histiocytes. However, the patient lacked multisystemic features of ECD, foamy histiocytes were absent, and BRAF V600E mutation was not detected. Considering the slow progression of illness over a 4 year period and radiological evidence suggestive of bone remodeling with an absence of aggressive features and severe narrowing of medullary canal in the lower limbs, the patient underwent molecular analysis for Ghosal Hemato Diaphyseal Dysplasia (GHDD). Results revealed homozygous p.Arg412Gln (exon 11) in the TBXAS1 gene, indicating an autosomal recessive pattern for this disease. Considering the anti-inflammatory effects of NSAIDs on the cyclooxygenase and its downstream metabolites, oral Aspirin 150 mg/day was initiated. Within 3 months of aspirin treatment, the patient's knee pain was resolved and the hemoglobin improved to 11 indicating a notable response. Patient was advised to undergo lifelong treatment with aspirin.

## Discussion

Genetic information was available in only a few cases of GHDD, with the founder variant p.Arg413Gln initially identified in a Pakistani family. Subsequently, 4 variants (c.1463T>C p.Leu488Pro; c.248T>C p.Leu83Pro; c.1444G>T p.Gly482Trp; c.1238G>A p.Arg413Glu) have been described with 2 Indian-origin variants, exhibiting distinct features [3,7]. Recently, several novel TBXAS1 variants have been identified using molecular analysis (whole-exome and next-genome sequencing) [4]. Our study presents a homozygous mutation in exon 11 of the TBXAS1 gene, specifically p.Arg412Gln, which has not been previously reported.

Patients typically present in childhood with characteristic bony changes and persistent normocytic anemia often requiring multiple transfusions [4]. However, in adults, symptoms usually appear as pain/discomfort in weight-bearing joints similar to our case or widening in the extremities [2]. These patients experience various hematological manifestations due to impaired hematopoiesis, ranging from mild myelophthisic anemia and thrombocytopenia to severe pancytopenia necessitating RBC transfusion [6,10]. The most characteristic feature is progressive normocytic normochromic anemia, whereas only 50% of patients present with thrombocytopenia [11].

Radiologically, GHDD is characterized by the expansion and sclerosis of the diaphysis of long bones, accompanied by the widening of medullary cavities and cortical hyperostosis [11] with skull base involvement occurring in certain cases [6]. PET-CT scan can be used for the diagnosis of hemato-oncologic conditions involving the bone marrow [12]. The bone marrow biopsy frequently reveals hypocellularity, and in some cases, myelofibrosis or decreased erythroid precursors [2,4].

Considering the immunological aspects of GHDD, involving the TBXAS1 gene dysregulation, patients were treated with prednisone of varying dosages, either in short courses titrated to the patient's response or in maintenance doses indefinitely for refractory cases [2,4,11]. However, no standard regimen has been designed to date [7]. Brown et al hypothesized that NSAIDs could be a safer first-line therapy for GHDD. TBXAS1 gene dysregulation increases prostaglandin H2, impairing osteoclastic activity, causing sclerosis, and bone marrow injury. NSAIDs inhibiting COX-1 and COX-2 prevent prostaglandin production, restoring hematopoiesis [4,[6], [7], [8],^13^]. Brown et al showed improvement in hematological parameters and inflammatory markers with the daily use of NSAIDs (Aspirin 325 mg or ibuprofen 30 mg/kg) [13]. Our study is a pioneering example of utilizing low-dose aspirin as a first-line therapy for GHDD. Timely diagnosis of GHDD is of utmost importance since treatment is a readily available over-the-counter drug and can effectively improve anemia and bone changes. This avoids the necessity for further transfusions [10].

## Conclusion

By analyzing radiological evidence alongside a high level of clinical suspicion, a diagnosis of adult-onset GHDD can be achieved. Timely diagnosis of GHDD is of utmost importance since treatment is a readily available over-the-counter drug. This case report serves to emphasize the significance of relying on radiological evidence when diagnosing late-onset genetic disorders.

## Author contributions

1. Diviya Bharathi Ravikumar-Drafted the entire manuscript, particularly contributed to introduction and case presentation.
2. Shreya Thungala-Identified the case, involved in introduction and table drafting.
3. Barath Prashanth Sivasubramanian-Drafted the entire manuscript, particularly contributed to case presentation and discussion.
4. Gopinath Srinivasan-Identified the case and reviewed of manuscript.
5. Abul Hasan Shadali Abdul Khader-Contributed to drafting the discussion.
6. Husna Qadeer-Contributed to drafting the abstract.
7. Viraj Panchal-Review of manuscript.
8. Vikram Samala Venkata-Review of manuscript.

## Patient consent

I am pleased to confirm that the patient has granted consent for the publication of their case. They understand and agree to the inclusion of their medical details in professional publications, with confidentiality being maintained.

## Disclosure

All authors agreed to the findings of the case.
